# Comparing Door-To-Balloon Time between ST-Elevation Myocardial Infarction Electrocardiogram and Its Equivalents

**DOI:** 10.3390/jcm11195547

**Published:** 2022-09-22

**Authors:** Youngchul Choi, Kiwook Kim, Joo Suk Oh, Hyun Ho Jeong, Jung Taek Park, Yeon Young Kyong, Young Min Oh, Se Min Choi, Kyoung Ho Choi

**Affiliations:** Department of Emergency Medicine, Uijeongbu St. Mary’s Hospital, College of Medicine, The Catholic University of Korea, 271 Cheonbo-ro, Uijeongbu-si 11765, Korea

**Keywords:** ST-elevation myocardial infarction, door-to-balloon time, acute coronary syndrome, coronary occlusion, emergency department, percutaneous coronary intervention

## Abstract

Background: In patients with ST-elevation myocardial infarction (STEMI) undergoing primary percutaneous coronary interventions (pPCI), longer door-to-balloon (DTB) time is known to be associated with an unfavorable outcome. A percentage of patients with acute coronary occlusion present with atypical electrocardiographic (ECG) findings, known as STEMI-equivalents. We investigated whether DTB time for STEMI-equivalent patients was delayed. Methods: This is a retrospective study including patients arriving at an emergency department with the acute coronary syndrome in whom emergent pPCI was performed. ECGs were classified into STEMI and STEMI-equivalent groups. We compared DTB time, with its components, between the groups. We also investigated whether STEMI-equivalent ECG was an independent predictor of DTB time delayed for more than 90 min. Results: A total of 180 patients were included in the present study, and 23 patients (12.8%) presented with STEMI-equivalent ECGs. DTB time was significantly delayed in patients with STEMI-equivalent ECGs (89 (80–122) vs. 81 (70–88) min, *p* = 0.001). Multivariable logistic regression analysis showed that STEMI-equivalent ECG was an independent predictor of delayed DTB time (odds ratio: 4.692; 95% confidence interval: 1.632–13.490, *p* = 0.004). Conclusions: DTB time was significantly delayed in patients presenting with STEMI-equivalent ECGs. Prompt recognition of STEMI-equivalent ECGs by emergency physicians and interventional cardiologists might reduce DTB time and lead to a better clinical outcome.

## 1. Introduction

Despite enormous advancements in medical technology in recent years, acute coronary syndrome (ACS) remains a challenge to health care professionals [1]. ST-segment elevation myocardial infarction (STEMI) is a type of ACS that requires immediate medical attention, and emergent reperfusion therapy is strongly recommended in such cases [2,3]. In STEMI patients, early primary percutaneous coronary intervention (pPCI) is the dominant treatment strategy [4,5]. Reducing the ischemic time from symptom onset to coronary reperfusion is crucial, and door-to-balloon (DTB) time, defined as the time duration from patient arrival at the emergency department (ED) to device placement on an infarct coronary artery, is frequently used as an indicator of adequate pPCI performance [6]. Previous studies have shown that delayed DTB time is associated with an unfavorable outcome in patients with STEMI [7,8,9,10,11,12,13]. Therefore, clinical guidelines recommend maintaining shorter ischemic time for these patients [2,3].

However, there is a possibility that many other factors can influence the DTB time [14]. A delay in electrocardiographic (ECG) interpretation might be one of the possible causes [15,16]. Some patients who arrive at the ED with an acute coronary occlusion present with atypical electrocardiographic findings that are inconsistent with the classical STEMI patterns [17,18]. Although current guidelines recommend performing prompt pPCI for these patients, the attending physicians might delay catheterization laboratory activation and the decision to perform pPCI if they are not familiar with atypical electrographic findings of STEMI. Therefore, the objective of this study was to investigate whether the atypical electrographic findings, also known as STEMI-equivalents, might be associated with delayed DTB time compared to classical STEMI findings.

## 2. Materials and Methods

### 2.1. Study Design

This was a single-centered retrospective chart study of ACS patients who arrived at a regional emergency center of an urban teaching hospital in South Korea, with the facilities of performing pPCI from January 2019 to December 2019. This study period was chosen to eliminate the potential effect of the worldwide COVID-19 outbreak starting in January 2020 [19]. Patients over 18 years of age showing symptoms and ECG findings consistent with acute myocardial infarction (AMI), according to the 4th Universal Definition of Myocardial Infarction [20], and who underwent catheterization laboratory activation and emergent pPCI at the hospital were included in this study. Patients were included in the study irrespective of the method of arrival at the ED. Exclusion criteria were: no STEMI or STEMI-equivalent findings in the ECG, delayed catheterization laboratory activation due to ischemic changes in subsequent ECGs, no evidence of coronary stenosis or occlusion by coronary angiography (e.g., AMI caused by vasospasm, myocardial infarction with non-obstructive coronary arteries, false positive ECG, etc.), pPCI failure, or application of extracorporeal membrane oxygenation (ECMO) in the ED before pPCI. This study was approved by the Institutional Review Board of Uijeongbu St. Mary’s Hospital (UC22RASI0067). The requirement of informed consent was waived due to the study’s retrospective nature.

### 2.2. Setting

During the study period, obtaining 12-lead ECG by prehospital emergency medical technicians was not within their scope of work, as per the law in South Korea. Although most of the emergency medical technicians were capable of obtaining and interpreting 12-lead ECGs, and many reported possible ST-segment elevations in 3-lead and/or 12-lead ECGs, they were neither eligible nor obliged to report ECG interpretations. Due to such limitations, prehospital catheterization laboratory activation was not applicable. Therefore, catheterization laboratory activation was performed by attending emergency physicians for all the study subjects.

After catheterization laboratory activation, the interventional cardiologist on duty reviewed the patient history and ECGs, performed bedside echocardiography, if needed, and made decisions regarding whether to perform emergent pPCI. Our hospital has one catheterization laboratory dedicated to coronary angiography. If this was unavailable, the patient was either transferred out or underwent thrombolytic therapy. Such cases were not included in this study because they did not meet the inclusion criteria.

### 2.3. Data Collection

Baseline characteristics, including age, sex, comorbidities, time and method of ED arrival, initial vital signs, laboratory and radiographic findings at admission, medication use before or after pPCI, event before performing pPCI, and in-hospital outcomes of the study participants were collected from their electronic medical records. ED presentation at working hours was defined as a patient visit from 8:30 AM to 17:00 PM from Monday to Friday, excluding national holidays. The left ventricular ejection fraction was obtained by transthoracic echocardiogram after pPCI by a cardiologist. The angiographic and procedural characteristics were obtained from the coronary angiography report written by the interventional cardiologist who performed the pPCI.

A single ECG machine (Pagewriter TC70; Philips, Amsterdam, The Netherlands) was used for all patients who visited the ED. All ECGs were obtained at a speed of 25 mm/s and amplification of 10 mm/mV. ECGs were interpreted and classified into two categories (STEMI and STEMI-equivalent) according to the 2017 European Society of Cardiology guidelines for STEMI [3]. STEMI was defined as ECG findings of at least two contiguous leads with new ST-segment elevation at the J point ≥ 2.5 mm in men < 40 years, ≥2 mm in men ≥40 years, or ≥1.5 mm in women in leads V_2_–V_3_ and/or ≥1 mm in any other leads. STEMI ECGs were classified as anterior (ST-segment elevation in leads V_1_–V_4_), inferior (ST-segment elevation in leads II, III, aVF), or lateral (ST-segment elevation in leads I, aVL, V_5_–V_6_). STEMI-equivalent was classified according to Table 1. It was defined as ECG finding of left bundle branch block (LBBB) or ventricular paced rhythm with positive modified Sgarbossa criteria [21], new- or presumably new-onset right bundle branch block (RBBB) without ST-segment elevation, isolated posterior myocardial infarction defined as ST-segment depression in leads V_1_–V_3_ and/or ≥0.5 mm ST-segment elevation in posterior leads (V_7_–V_9_), or ST-segment elevation in lead aVR with ST-segment depression ≥ 1 mm in eight or more surface leads. A trained investigator (K.K.) who was blinded to patient information reviewed all the ECGs independently.

Time variables were collected and defined as follows: time from arrival at the ED to ECG test (Door-to-ECG (DTE) time), time from ECG test to catheterization laboratory activation (ECG-to-activation (ETA) time), time from catheterization laboratory activation to arrival at the catheterization laboratory (activation-to-laboratory (ATL) time), and time from arrival at the catheterization laboratory to balloon dilatation or thrombus aspiration at the culprit artery (laboratory-to-balloon (LTB) time) [14]. The DTB time was defined as a composite of all time variables. The primary outcomes of this study were the DTB time with its components. The secondary outcome was the occurrence of in-hospital major adverse cardiovascular events (MACEs), defined as a composite of in-hospital repeat revascularization and congestive heart failure with pulmonary edema and death.

### 2.4. Statistical Analysis

Baseline, angiographic, procedural characteristics, and time variables were compared between STEMI and STEMI-equivalent groups. Categorical variables are presented as numbers (percentages). They were compared by chi-squared tests. Continuous variables were first tested for normal distribution using Kolmogorov–Smirnov tests. Normally distributed data are presented as means ± standard deviations. They were compared using Student’s *t*-tests. Non-normally distributed data are presented as medians (interquartile range (IQR)). They were compared using Mann–Whitney *U* tests.

Multivariable logistic regression analysis was performed to determine whether STEMI-equivalent ECG was independently associated with DTB time delayed for longer than 90 min. Possible confounding factors such as age, female sex, ED presentation at working hours, transfer in from other hospitals, cardiac arrest before pPCI, radial artery cannulation for pPCI compared to femoral artery cannulation, and thrombus aspiration before balloon dilatation during pPCI were selected as variables. Univariable logistic regression analyses were performed for each variable. Variables with a *p*-value < 0.05 were entered into multivariable logistic regression analysis. To identify independently associated factors of in-hospital MACE, age, female sex, cardiac arrest before pPCI, three-vessel disease, left main artery disease, pre-pPCI thrombolysis in myocardial infarction (TIMI) grade flow of 0, STEMI-equivalent ECG, and DTB time were selected as variables. Univariable and multivariable logistic regression analyses were performed as described above. All statistical analyses were performed using MedCalc Statistical Software version 20.110 (MedCalc Software, Ostend, Belgium). A two-tailed *p*-value < 0.05 was considered statistically significant.

## 3. Results

**Figure 1:**
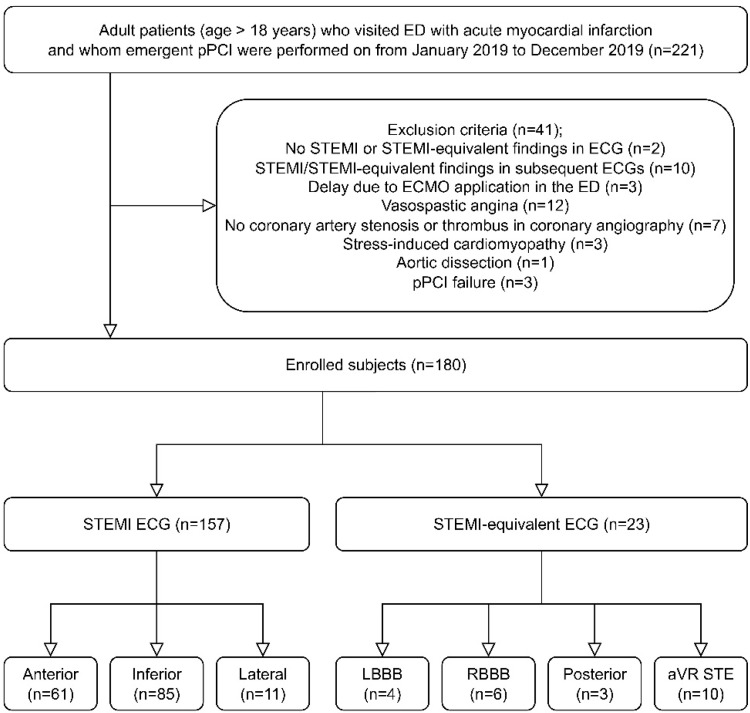
Flow chart of this study. Abbreviations: ECG, electrocardiogram; ECMO, extracorporeal membrane oxygenation; ED, emergency department; LBBB, left bundle branch block; pPCI, primary percutaneous coronary interventions; RBBB, right bundle branch block; STE, ST-segment elevation; STEMI, ST-segment elevation myocardial infarction.

Figure 1 depicts the design of this study. During the study period, 221 AMI patients visited our ED and underwent emergent pPCI. After excluding 41 patients who met the exclusion criteria, a total of 180 patients were eventually enrolled. Of these study subjects, 23 (12.8%) presented with STEMI-equivalent ECGs (4 with LBBB or ventricular paced rhythm with positive modified Sgarbossa criteria, 6 with new- or presumably new-onset RBBB with ST-segment elevation, 3 with isolated posterior myocardial infarction, and 10 with ST-segment elevation in aVR).

The baseline characteristics of the two groups are described in Table 2. Patients presenting with STEMI-equivalent ECGs had significantly faster heart rate (85 (75–107) bpm vs. 77 (66–89) bpm, *p* = 0.044) and higher serum creatinine (1.36 (1.18–1.69) mg/dL vs. 1.13 (0.97–1.31) mg/dL, *p* = 0.002) than patients presenting with STEMI ECGs. The STEMI-equivalent group was also more likely to suffer from cardiac arrest before pPCI (43.5% vs. 10.2%, *p* < 0.001). There were no statistically significant differences in other baseline characteristics between the two groups. The angiographic and procedural characteristics are described in Table 3. Pre-pPCI TIMI grade flow by coronary angiography was significantly higher in patients presenting with STEMI-equivalent ECGs (1 (0–2) vs. 0 (0–1), *p* = 0.007). The radial artery cannulation approach was significantly more common in STEMI patients (73.2% vs. 47.8%, *p* = 0.013).

Time variables were compared between the groups. ETA time (4 (2–12) vs. 2 (1–5) min, *p* = 0.036) and ATL time (63 (47–82) vs. 49 (40–54) min, *p* = 0.001) were significantly delayed in patients presenting with STEMI-equivalent ECGs than in STEMI patients. DTB time was also significantly delayed in patients with STEMI-equivalent ECGs (89 (80–122) vs. 81 (70–88) min, *p* = 0.001).

Univariable and multivariable logistic regression analyses were performed to identify independent predictors of DTB time delayed for more than 90 min. Results are described in Table 4. DTB time was delayed for more than 90 min in a total of 28 (15.6%) patients. Univariable logistic regression analysis showed that STEMI-equivalent ECG was a predictor of delayed DTB time (odds ratio (OR): 7.549, 95% confidence interval (CI): 2.889–19.728, *p* < 0.001). After adjusting for possible confounding factors, STEMI-equivalent ECG remained an independent predictor of delayed DTB time (OR: 4.692, 95% CI: 1.632–13.490, *p* = 0.004). Cardiac arrest before performing pPCI (OR: 3.511, 95% CI: 1.184–10.411, *p* = 0.024) was another independent predictor of delayed DTB time. Although presenting with STEMI-equivalent ECGs was not independently associated with in-hospital MACE (OR: 1.592, 95% CI: 0.522–4.852, *p* = 0.414), longer DTB time was identified as an independent predictor of in-hospital MACE (OR: 1.020, 95% CI: 1.002–1.038, *p* = 0.033) (Table 5).

## 4. Discussion

To the best of our knowledge, this is the first study to analyze the influence of atypical ECG presentation on the DTB time of AMI patients requiring emergent pPCI. Our study showed that STEMI-equivalent ECG might be one of the factors affecting DTB time. Compared to patients with STEMI ECGs, ETA and ATL time were significantly delayed in patients presenting with STEMI-equivalent ECGs, which seemed to contribute to a delay in DTB time. Multivariable logistic regression analysis also showed that STEMI-equivalent ECG was an independent factor of DTB time delayed for more than 90 min, which is known to be associated with an unfavorable outcome in the study population. Our study also corroborated with previous studies in that delayed DTB time was an independent predictor of in-hospital MACE [7,8,10,11,13], indicating that the principle of valuing every minute for AMI patients might also be applied to patients with STEMI-equivalent ECGs, and timely reperfusion would be beneficial for this study population.

One important factor that might influence time delay in patients with STEMI-equivalent ECGs may be the unfamiliarity of healthcare providers with these ECGs. Compared to evaluating STEMI EEGs, which is universally taught to all types of healthcare providers, STEMI-equivalent ECGs are likely to be less familiar among emergency physicians and interventional cardiologists. Emergency physicians make decisions for catheterization laboratory activation. Delayed activation might lead to increased ETA time. Interventional cardiologists make decisions for emergent pPCI. Deferred reperfusion might influence ATL time. Since our study showed that ETA and ATL time were delayed in patients with STEMI-equivalent ECGs, it is hypothesized that unfamiliarity with STEMI-equivalent ECGs might have played an important role in DTB time delay. These findings corroborate with some previous studies showing that patients presenting with atypical ECGs are frequently mistaken as non-STEMI ECGs, thereby leading to delayed reperfusion [22]. One of the reasons for this unfamiliarity might be due to the rarity of STEMI-equivalent ECGs, which are known to be much less prevalent [23,24,25].

Another possible barrier to prompt pPCI might be the complexity of STEMI-equivalent ECG interpretation. Each entity owns various methods for the confirmation of a diagnosis. For example, Sgarbossa criteria are suggested by the European Society of Cardiology to detect acute coronary occlusion in LBBB and ventricular-paced rhythm [3]. These require physicians to evaluate ECGs according to three different categories [23]. The more recently developed Smith-modified Sgarbossa criteria [21] and Barcelona criteria [26] are slight modifications of the original Sgarbossa criteria. Interpreting ECGs according to these criteria will inevitably take more time than interpreting classical STEMI ECGs. A better knowledge of these criteria may help to distinguish STEMI-equivalent ECGs, leading to prompt catheterization laboratory activation and reperfusion. Isolated posterior myocardial infarction also requires attending physicians to recognize the typical ECG findings. Moreover, to confirm the diagnosis, additional ECGs, including posterior leads (V_7_–V_9_), must be obtained [27]. Although this would not cause a significant delay in many cases, it can be troublesome to obtain posterior leads in certain situations, such as intubated patients or patients in a coma.

Issues regarding ECGs with ST-segment elevation in lead aVR with ST-segment depression in multiple surface leads are even more complicated. Several studies have shown that these findings are associated with left main coronary occlusion [28,29]. However, not every ST-segment elevation in lead aVR is associated with coronary occlusion, and it may be present in triple-vessel disease or diffuse subendocardial ischemia [30]. Since diffuse subendocardial ischemia can be caused by any critical condition that leads to supply and demand mismatch [31], a large portion of patients presenting with ST-segment elevation in aVR might have no acute coronary occlusion. A recent study showed that only 10.1% of patients with ST-segment elevation in aVR who had undergone coronary angiography had an acute culprit coronary lesion [25]. Therefore, patients with ST-segment elevation in aVR might require a more thorough investigation than patients with classical STEMI ECGs. Diagnostic tools such as bedside echocardiography might help diagnose acute coronary occlusion [32]. However, these measures are time-consuming, and they will inevitably delay the decision for reperfusion. Interpretation of RBBB ECGs is similarly challenging because there are no diagnostic criteria to help diagnose acute coronary occlusion for these ECGs. Studies regarding this topic are extremely limited. Due to these limitations, physicians facing RBBB ECGs may need to utilize similar measures to those mentioned above. Developing diagnostic criteria to distinguish acute coronary occlusion for RBBB may be helpful, and future studies on this topic are warranted.

Several factors are known to affect DTB time [14]. In our study, radial artery approach was not an independent factor for delayed DTB time, which corroborates with a previous study [33]. Although femoral artery cannulation was more common in patients with STEMI-equivalent ECGs, it did not affect DTB time. Meanwhile, cardiac arrest before pPCI was an independent factor for delayed DTB time, which has never reported before, to the best of our knowledge. A cardiac arrest requires time-consuming measures during resuscitation and the post-resuscitation period. Moreover, it is difficult to distinguish patients with acute coronary occlusion as the cause of arrest from patients with other causes of arrest because many cardiac arrest patients with acute coronary occlusion do not present with STEMI findings after the return of spontaneous circulation [34]. Recent trials have also shown that performing emergent coronary angiographies for all out-of-hospital cardiac arrest patients without STEMI ECGs is not beneficial compared to usual care [35,36], which might make interventional cardiologists hesitant to perform emergent coronary angiography. Since STEMI-equivalent ECGs are known to represent acute coronary occlusion, patients with these ECGs after cardiac arrest may require additional medical attention, and early coronary angiography may need to be considered.

There has been concern that a significant portion of patients not presenting with STEMI ECGs show an acute coronary occlusion in coronary angiography [37,38,39,40]. A recent study compared STEMI ECGs and non-STEMI ECGs for patients with acute coronary occlusion on coronary angiography and found that ED arrival to catheterization time is significantly delayed in patients with non-STEMI ECGs, while adverse outcomes are similar between the two groups [41]. This was in part similar to our study in that both studies showed delayed DTB time in patients with non-STEMI ECGs compared to STEMI ECGs, and that earlier diagnosis and treatment for these patients might be highly valuable. Compared to the previous study, which simply compared STEMI and non-STEMI ECGs, our study focused more on STEMI-equivalent ECGs, which may be more meaningful in clinical practice because these specific ECGs are more likely to represent acute coronary occlusion.

This study has several limitations. First, this was a single-centered retrospective study based on a chart review. Although we tried to be as comprehensive as possible when selecting variables, this study was still prone to potential unidentified confounders and selection bias. Additionally, the number of study participants was relatively small, since there were only 23 patients with STEMI-equivalent ECGs. Results might need to be validated by multi-center, prospective studies with an adequate sample size. Second, we did not use the more recent concept of first medical contact to reperfusion time in our study, which is the preferred quality of care indicator, according to recent guidelines [3]. As mentioned in the Materials and Methods section, emergency medical technicians were not obliged to report ECG findings to emergency physicians. In addition, prehospital catheterization laboratory activation was inapplicable in South Korea. Whether first medical contact to reperfusion time is also delayed in patients with STEMI-equivalent ECGs might require further investigation. Third, there might be other types of STEMI-equivalent ECGs that are not addressed in our study. de Winter’s T waves [42], the South African Flag sign [43], and the Aslanger pattern [44] are some of the ECG findings known to represent acute coronary occlusion. These findings were not included in our study because none of the study population presented with such ECG findings. This might be due to the unfamiliarity of health care workers with these findings; thus, the catheterization laboratory might not have been activated for patients presenting with these ECGs. When future studies are designed, these findings also need to be addressed.

## 5. Conclusions

ETA, ATL, and DTB times were significantly delayed in patients presenting with STEMI-equivalent ECGs. Delayed DTB time was independently associated with in-hospital MACE. Prompt recognition of STEMI-equivalent ECGs by emergency physicians and interventional cardiologists might be crucial to reduce DTB and overall ischemic time, which may lead to a better clinical outcome in patients presenting with atypical ECG findings.

## Figures and Tables

**Table 1:** Definition and classification of STEMI-equivalent electrocardiogram according to the 2017 STEMI guidelines from the European Society of Cardiology [3].

Left bundle branch block	Criteria that can be used to improve the diagnostic accuracy of STEMI in LBBB.
	* Concordant ST-segment elevation ≥ 1 mm in leads with a positive QRS complex.
	* Concordant ST-segment depression ≥ 1 mm in V_1_–V_3_.
	* Excessive relative discordant ST-segment elevation in any single lead, defined by ST-segment elevation ≥ 25% of preceding S-wave depth [21].
Ventricular paced rhythm	During right ventricular pacing, the ECG also shows LBBB, and the above rules apply to the diagnosis of myocardial infarction; however, they are less specific.
Right bundle branch block	The presence of RBBB may confound the diagnosis of STEMI.
Isolated posterior myocardial infarction	Isolated ST-segment depression ≥ 0.5 mm in leads V_1_–V_3_ and/or ST-segment elevation ≥ 0.5 mm in posterior chest wall leads V_7_–V_9_.
ST-segment elevation in aVR	ST-segment depression ≥ 1 mm in eight or more surface leads, coupled with ST-segment elevation in aVR and/or V_1_, suggests left main or left main equivalent coronary obstruction, or severe three-vessel disease.

Abbreviations: ECG, electrocardiogram; LBBB, left bundle branch block; RBBB, right bundle branch block; STEMI, ST-segment elevation myocardial infarction.

**Table 2:** Baseline characteristics of patients presenting with STEMI or STEMI-equivalent electrocardiograms.

	STEMI	STEMI-Equivalent	*p*
	*n* = 157	*n* = 23	
Age, y	62 (53–73)	69 (55–81)	0.168
Female sex	32 (20.4)	7 (30.4)	0.276
Body mass index, kg/m^2^	24.0 ± 3.0	24.3 ± 2.9	0.637
Comorbidities			
Hypertension	68 (43.3)	12 (52.2)	0.426
Diabetes mellitus	37 (23.6)	8 (34.8)	0.247
Known previous coronary artery disease	14 (8.9)	4 (17.4)	0.207
History of smoking	70 (44.6)	8 (34.8)	0.377
ED presentation at working hours	63 (40.1)	8 (34.8)	0.625
Transferred in from another hospital	48 (30.6)	6 (26.1)	0.662
Initial vital signs			
Mean arterial pressure, mmHg	88.1 ± 17.6	84.8 ± 22.1	0.414
Heart rate, bpm	77 (66–89)	85 (75–107)	0.044
Body temperature, °C	36.0 (36.0–36.2)	36.0 (36.0–36.0)	0.109
Oxygen saturation, %	98 (96–100)	99 (95–100)	0.596
Initial laboratory findings			
Hemoglobin, g/dL	15.0 (13.4–16.0)	14.4 (12.3–15.5)	0.152
Serum creatinine, mg/dL	1.13 (0.97–1.31)	1.36 (1.18–1.69)	0.002
Troponin T, ng/mL	0.042 (0.016–0.484)	0.114 (0.048–0.422)	0.095
Creatine kinase, U/L	163 (101–466)	211 (124–329)	0.416
Creatine kinase-myocardial band, ng/mL	3.61 (2.14–13.97)	7.64 (3.94–17.33)	0.079
Pulmonary edema in initial chest X-ray	62 (39.5)	10 (43.5)	0.716
Left ventricular ejection fraction, %	52.5 (45.0–58.0)	54.0 (45.0–60.0)	0.935
Medication use			
Aspirin	154 (98.1)	22 (95.7)	0.460
Clopidogrel	37 (23.6)	7 (30.4)	0.475
Prasugrel	9 (5.7)	1 (4.3)	0.787
Ticagrelor	117 (74.5)	15 (65.2)	0.347
Heparin	79 (50.3)	10 (43.5)	0.541
Glycoprotein IIb/IIIa inhibitor	50 (31.8)	5 (21.7)	0.327
Trimetazidine	15 (9.6)	2 (8.7)	0.896
Cardiac arrest before pPCI	16 (10.2)	10 (43.5)	<0.001

Data are expressed as number (%), mean ± standard deviation, or median (interquartile range). Abbreviations: ED, emergency department; pPCI, primary percutaneous coronary intervention; STEMI, ST-segment elevation myocardial infarction.

**Table 3:** Angiographic and procedural characteristics of patients presenting with STEMI or STEMI-equivalent electrocardiograms.

	STEMI	STEMI-Equivalent	*p*
	*n* = 157	*n* = 23	
Angiographic characteristics			
Number of narrowed vessels			0.195
Single-vessel disease	95 (60.5)	11 (47.8)	
Double-vessel disease	38 (24.2)	5 (21.7)	
Triple-vessel disease	24 (15.3)	7 (30.4)	
Culprit artery			<0.001
Left main artery	2 (1.3)	3 (13.0)	
Left anterior descending artery	76 (48.4)	8 (34.8)	
Right coronary artery	69 (43.9)	6 (26.1)	
Left circumflex artery	10 (6.4)	6 (26.1)	
Pre-pPCI TIMI grade flow of culprit artery	0 (0–1)	1 (0–2)	0.026
Positive spasm provocation test	2 (1.3)	0 (0.0)	0.587
Procedural characteristics			
Radial artery cannulation	115 (73.2)	11 (47.8)	0.013
Thrombus aspiration before balloon dilatation	15 (9.6)	2 (8.7)	0.896
Time variables			
Door-to-ECG (DTE) time, min	4 (1–7)	2 (1–7)	0.641
ECG-to-activation (ETA) time, min	2 (1–5)	4 (2–12)	0.036
Activation-to-laboratory arrival (ATL) time, min	49 (40–54)	63 (47–82)	0.001
Laboratory arrival-to-balloon (LTB) time, min	22 (17–27)	22 (19–29)	0.473
Door-to-balloon (DTB) time, min	81 (70–88)	89 (80–122)	0.001

Data are expressed as number (%) or median (interquartile range). Abbreviations: ECG, electrocardiogram; pPCI, primary percutaneous coronary intervention; STEMI, ST-segment elevation myocardial infarction; TIMI, thrombosis in myocardial infarction.

**Table 4:** Univariable and multivariable logistic regression analyses for predicting delayed door-to-balloon time for more than 90 min.

	Crude			Adjusted		
	OR	95% CI	*p*	OR	95% CI	*p*
STEMI-equivalent ECG	7.549	2.889–19.728	<0.001	4.692	1.632–13.490	0.004
Age, y	1.021	0.990–1.053	0.192			
Female sex	0.984	0.369–2.625	0.974			
ED presentation at working hours	0.688	0.292–1.619	0.391			
Transferred in from another hospital	0.591	0.225–1.552	0.285			
Cardiac arrest before pPCI	7.393	2.921–18.713	<0.001	3.511	1.184–10.411	0.024
Radial artery cannulation for pPCI	0.250	0.109–0.575	0.001	0.448	0.170–1.184	0.105
Thrombus aspiration before balloon dilatation during pPCI	2.536	0.817–7.872	0.107			

A *p*-value of <0.05 was included in the multivariable logistic regression model. Abbreviations: CI, confidence interval; ECG, electrocardiogram; ED, emergency department; OR, odds ratio; pPCI, primary percutaneous coronary intervention; STEMI, ST-segment elevation myocardial infarction.

**Table 5:** Univariable and multivariable logistic regression analyses for predicting in-hospital major adverse cardiovascular events.

	Crude			Adjusted		
	OR	95% CI	*p*	OR	95% CI	*p*
STEMI-equivalent ECG	3.947	1.601–9.726	0.003	1.592	0.522–4.852	0.414
Door to balloon time, min	1.028	1.012–1.045	0.001	1.020	1.002–1.038	0.033
Age, y	1.061	1.031–1.093	<0.001	1.070	1.033–1.109	<0.001
Female sex	2.218	1.039–4.734	0.040	1.000	0.402–2.491	1.000
Cardiac arrest before pPCI	3.667	1.552–8.663	0.003	4.508	1.473–13.792	0.008
Triple-vessel disease	2.136	0.943–4.839	0.069			
Left main artery disease	3.047	0.593–15.658	0.182			
Pre-pPCI TIMI grade flow of 0	1.284	0.615–2.677	0.506			

A *p*-value of <0.05 was included in the multivariable logistic regression model. Abbreviations: CI, confidence interval; ECG, electrocardiogram; OR, odds ratio; pPCI, primary percutaneous coronary intervention; STEMI, ST-segment elevation myocardial infarction; TIMI, thrombolysis in myocardial infarction.

## Data Availability

The data analyzed during the study are available upon reasonable request from the corresponding author.
